# LED Lights Promote Growth and Flavonoid Accumulation of *Anoectochilus roxburghii* and Are Linked to the Enhanced Expression of Several Related Genes

**DOI:** 10.3390/plants9101344

**Published:** 2020-10-12

**Authors:** Do Thi Gam, Phan Hong Khoi, Pham Bich Ngoc, Ly Khanh Linh, Nguyen Khac Hung, Phan Thi Lan Anh, Nguyen Thi Thu, Nguyen Thi Thu Hien, Tran Dang Khanh, Chu Hoang Ha

**Affiliations:** 1 Institute of Biotechnology, Vietnam Academy of Science and Technology (VAST), 18 Hoang Quoc Viet, Nghia Do, Cau Giay, Hanoi 100000, Vietnam; honggam@htd.vast.vn (D.T.G.); pbngoc@ibt.ac.vn (P.B.N.); lykali139@gmail.com (L.K.L.); hungkhacibt@gmail.com (N.K.H.); nguyenhien2111@gmail.com (N.T.T.H.); 2 Center for High Technology Development, Vietnam Academy of Science and Technology (VAST), 18 Hoang Quoc Viet, Nghia Do, Cau Giay, Hanoi 100000, Vietnam; phkhoi@htd.vast.vn (P.H.K.); lananh52cnsh@gmail.com (P.T.L.A.); nminhthu02@gmail.com (N.T.T.); 3 Vietnam Academy of Science and Technology (VAST), Graduate University of Science and Technology, 18 Hoang Quoc Viet, Nghia Do, Cau Giay, Hanoi 100000, Vietnam; 4 Agricultural Genetics Institute, Pham Van Dong, North Tu Liem, Hanoi 123000, Vietnam; tdkhanh@vaas.vn; 5 Center for Agricultural Innovation, Vietnam National University of Agriculture, Hanoi 131000, Vietnam

**Keywords:** *Anoectochillus roxburghii*, chalcone synthase, flavonol synthase, flavonoid, LED light

## Abstract

*Anoectochilus roxburghii* is a wild edible species and has been traditionally used for a wide range of diseases in many countries. Our research aims to find the optimal light-emitting diode (LED) lighting conditions to improve the growth and development of *A. roxburghii* seedling at the acclimation stage. Two-month-old explants were cultured under the various lighting conditions including red (R), blue (B), BR (one blue: four red), BRW151 (one blue: five red: one white), BRW142 (one blue: four red: two white), and fluorescent lamp (FL). The results showed that the lighting conditions not only affect the growth and morphology of plants but also the accumulation of total flavonoids. Single wavelengths (B or R LED) inhibited the growth and secondary biosynthesis of *A. roxburghii*, while the BR LED showed an enhancement in both growth and biomass accumulation. *A. roxburghii* plants were grown under BR LED light has average plant height (7.18 cm), stem diameter (17.6mm), number of leaves (5.78 leaves/tree), leaf area (4.67 cm^2^), fresh weight (0.459 g/tree), dry matter percentages (11.69%), and total flavonoid (1.811 mg/g FW) is considered to be superior to FL lamps and other LEDs in the experiment. This indicates that both blue and red wavelengths are required for the normal growth of *A. roxburghii*. To learn more about how light affects flavonoid biosynthesis, we evaluated the expression of genes involved in this process (*pal*, *chs*, *chi*, and *fls*) and found that BR LED light enhances the expression level of *chi* and *fls* genes compared to fluorescent lamps (1.18 and 1.21 times, respectively), leading to an increase in the flavonoid content of plant. Therefore, applying BR LED during in vitro propagation of *A. roxburghii* could be a feasible way to improve the medicinal value of this plant.

## 1. Introduction

*Anoectochilus roxburghii* (Orchidaceae) is one of the most valuable medicinal plants and is mainly distributed in Asian countries including Vietnam, India, Japan, Taiwan, and China. *A. roxburghii* is a wild edible herb and has been traditionally used for ornamental and a wide range of ailments in many countries. All parts of *A. roxburghii* have a long tradition use as a folk medicine to treat various ailments, with indications from one country to the other. In China, the whole plant has been used in various dosage forms such as oral liquid, spray, capsules, and its mixture to treat hepatitis, hyperliposis, tumor, chronic hepatitis B, diabetes [1,2,3,4,5] and nephritis, cystitis, myasthenia gravis, rheumatoid arthritis, febrile convulsion, cough-variant, and snakebite [6]. In Taiwan, all plant parts are frequently used as a tonic and treat hypertension, lung disease, kidney deficiency [7], cancer, and other incurable diseases [4]. The entire plant possesses various pharmacological activities such as antidiabetics, anti-inflammatory, protection of liver and renal, antineoplastic, immunomodulatory, and antioxidant activities [4]. Moreover, some clinical applications of this plant showed good efficacy on the type 2 diabetes mellitus [8], chronic hepatitis B [9], cough-variant asthma [10], hyperuricemia [11], hand foot and mouth disease [12], tic-coprolalia syndrome [13], and helicobacter pylori infection [14]. 

Recently, Ho et al. [15] reported that *A. roxburghii* water extract showed effects of antitumor, hypoglycemic, anti-HBV, and hypotensive. *A. roxburghii* extracts showed a high anti-inflammatory effect on xylene-induced mice [15]. It also inhibits the production of inflammatory mediators and enhances the production of anti-inflammatory cytokines [16]. Especially, *A. roxburghii* extracts significantly promote hepatoprotective activity in experimental mice as well as reduce the formation of early liver fibrosis [17]. *A. roxburghii* can decrease liver injury consequences via inhibiting liver and spleen enlargement [18]. The major compounds responsible for the liver protection effects possibly are flavonoids, which proved to be involved in the anti-oxidation activities including the removal of free radicals in liver cells, inhibition of lipid peroxidation, and stabilization of cell membranes’ processes [19,20]. Flavonoids belong to polyphenol, one of the most important groups’ secondary metabolites in plants. Polyphenol compounds are responsible for plant defenses against both biotic and abiotic stresses and even light irradiation [21]. Some major chemical constituents belonging to flavonoids, triterpenes, polysaccharides, alkaloids, nucleosides, steroids, glycosides, volatile compounds, and organic acids have been isolated and identified from the different parts of this plant. Many of them have been considered as the bioactive compounds related to the pharmacological activities such as antioxidant, antidiabetic, anti-inflammatory, antiviral, antilipemic, abirritant, sedative, liver and renal protectives, immunomodulatory, and antineoplastic effects [4]. 

Light is an indispensable environmental factor for plant growth and development. The application of artificial light is more useful than using chemicals to manage plant architecture and simultaneously reduce environmental impacts [22]. The prompt development of lighting technologies using light-emitting diodes (LEDs) has led to increasing the application of this technology for lighting in closed horticultural systems [23]. Various effects of LED light on morphology and development were observed in many plant subjects. Numerous studies showed that a combination of red and blue light at the appropriate ratio is more necessary for plant growth and development than a monochromatic red or blue LED light [24,25,26]. In addition, the characteristics of LED (light wavelength, intensity, and light duration) also significantly affect the accumulation of metabolites in plants [27]. An increase in primary and secondary compounds such as sugars, starches, vitamin C, proteins, and polyphenols, etc., was obviously seen in red or blue LEDs compared with fluorescent lamp [28]. Monochromatic red LED stimulated anthocyanin accumulation in *Malus domestica* Borkh more significantly than monochromatic blue LED, which has been identified as due to an increased expression of anthocyanin synthesis gene (*MdMYB10* and *MdUFGT* gene) under the influence of the red LED [29]. The phenol content, total isoflavones, and antioxidant capacity of soybean germ when grown under LED blue light (470 nm) were also higher than those of LED green (530 nm) and florescent light [30]. LED blue light (440–540 nm, peak wavelength of 469 nm) had a positive impact on *A. roxburghii* [31]. After 40 days of planting under LED Blue light, the harvested *A. roxburghii* plants had significantly higher biomass, chlorophyll content, and secondary metabolites (flavonoids and total polyphenols) compared with the non-illuminated formula and other LED formulas in the experiment [31]. The lowest total flavonoid content was in the formula yellow light (540–620 nm, peak wavelength 592 nm), while the total polyphenol content was the lowest in the green light treatment (460–600 nm, peak wavelength 519 nm) [31]. The green LED (510 nm), yellow LED (595 nm), and LED combination of red LED with white LED also increased the antioxidant properties and anthocyanin accumulation of many different crops [28]. The coordination ratio of blue and red LEDs less than 1 is optimal for anthocyanins accumulation in plants, whereas for flavonols accumulation, the amount of blue light can be reduced to zero depending on the specific phenolic compounds and specific plant types [32].

Flavonoids are synthesized via the phenylpropanoid metabolic pathway, which is regulated by numerous enzymes such as *pal*, *chs*, *chi*, and *fls* etc. These enzymes are induced by light conditions in both direct and indirect mechanisms [33,34]. Some recent reports showed that each plant species has often independently absorbed a specific light requirement for its maximum secondary metabolites including flavonoids. For example, a high amount of flavonoids of *Zingiber officinale* [35], *Lithocarpus litseifolius* [36], and *Labisa pumilar* [37] were accumulated under lower light intensity, while soybean (*Glycine max*) yielded a high amount of flavonoids under high light intensity [38].

Due to its high valuable medicinal properties and significant beneficial effects on human health and negligible side effects, *A. roxburghii* is being overexploited in nature and largely depleted. This plant was listed in the Red Book in many countries, including Vietnam, as an endangered species that is urgently conserved and protected. Much worldwide effort has been focused on *A. roxburghii* research and its products such as tissue culture and cultivation techniques. However, the wild *A. roxburghii* plants are often degenerated due to its specific growth conditions, delayed germination, and growth rates [4,39]. Therefore, to meet increasing the global market demands for its herbal and functional food properties as well as to conserve and protect the wild resource of this species, it is an imperative work to simultaneously improve both yield and secondary metabolites of this plant. To the best of our knowledge, no studies are available so far on the responses of various LED light sources on the gene expression activities and flavonoids accumulation of *A. roxburghii* in the acclimatization stage during in vitro propagation. Therefore, the present study aimed to evaluate the effects of using the various LED lights on the growth and morphological characteristics, production of total flavonoid contents, and expressional characteristics of several flavonoids-synthesis-related genes of this medicinal plant.

## 2. Results

### 2.1. Effects of LED Light Treatments on Growth and Morphological Characteristics 

**Figure 1:**
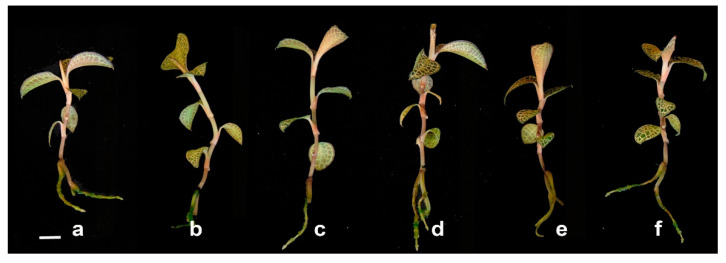
Effects of different light conditions on the *A. roxburghii* morphology; (**a**): fluorescent light; (**b**): red; (**c**): blue; (**d**): BR (one blue: four red); (**e**): BRW151 (one blue: five red: one white); (**f**): BRW142 (one blue: four red: two white). Scale bars = 1 cm.

**Figure 2:**
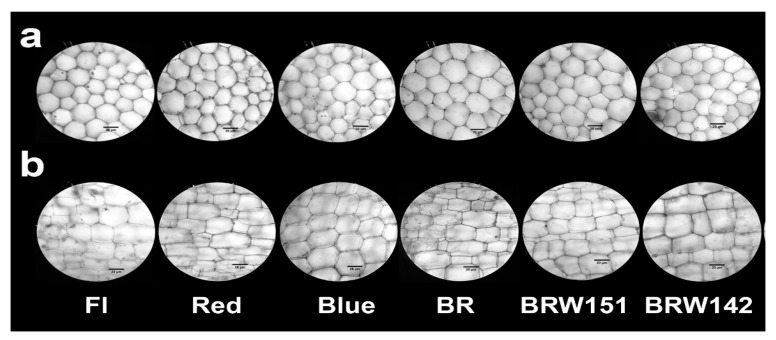
Effects of light conditions on *A. roxburghii* stem cell anatomy. Cross-sections (**a**) and longitude sections (**b**) of 2nd node of plants cultured under Fluorescent and LED lights (red, blue, BR, BRW151, and BRW142).

**Figure 3:**
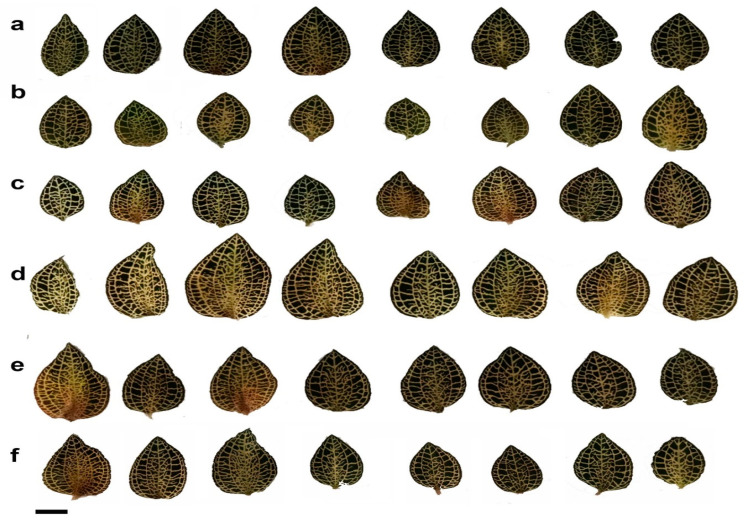
Effects of light condition on *A. roxburghii* leaves area. (**a**): fluorescent light; (**b**): red; (**c**): blue; (**d**): BR; (**e**): BRW151; (**f**): BRW142. Scale bars = 1 cm.

In this study, the different treatments of LEDs influenced the growth and morphological characteristics as well as cell anatomy of *A. roxburghii* were investigated. The average height of *A. roxburghii* was higher in the red, blue, and mixed BR treatments than in FL and BRW151 or BRW142 light (Figure 1; Table 1). The red light caused the most inhibitory effects on the stem diameter, while no significant difference in stem diameter was detected in all light treatments. They induced smaller and weaker stem diameter than under the fluorescent or other LED lights (Table 1). The highest number of leaves per plant was observed in BR (5.87 leaves), followed by BRW142 (5.8 leaves) and blue treatment (5.53 leaves). There were no significant differences between the red and BRW151 to compare with the FL (Table 1). Interestingly, the highest cell width was identified in blue treatment (36.87 µm), the next was BRW151 (33.11 µm), and the lowest was BR (30.31 µm). Contrarily, the cell width was inhibited under the red and BRW142 treatments. The highest cell length was in the red and BR by 44.33 µm and 44.2 µm, respectively. Meanwhile, the blue light remarkably suppressed the cell length and no significant difference was identified in BRW151 and BRW142 (Table 1, Figure 2). The leaf area was greater in the BR treatment (4.67 cm^2^) than the other light treatments. Nevertheless, there was no significant difference in leaf area among the blue, BRW151, and BRW142 treatments. Only the red light induced an inhibitory effect on the leaf area (1.99 cm^2^) in comparison with the FL treatment (3.36 cm^2^) (Table 1; Figure 3). The number of roots per plant was significantly increased in the blue and BRW142, while the other treatments showed negligible enhancement. The highest average of root length was in BRW142, and no significant difference was found in three treatments of red, BR, and BRW151.

The stem cell anatomy of this plant was significantly affected by the LEDs. Specifically, under the blue light, the highest cell width value was detected (36.87 µm), followed by BRW151 (33.11 µm) and BR (30.31 µm), while the lowest value of cell width was found under the red light (25.37 µm) and BRW142 (27.64 µm), which was lower than that of FL (28.58 µm). Interestingly, remarkable differences in cell length were found under the red and BR lights of which red light immensely promoted cell development in longitude direction; whereas, the blue light strongly inhibited the development (Table 1, Figure 2b). Compared to the control (FL) light, leaf area was dramatically reduced under the red light treatment, whilst the blue light, BRW151, and BRW142 exerted no significant effects. However, only BR was found to strikingly enhance the leaf area (Figure 3). The decreases in leaves enlargement significantly influenced the total biomass accumulation, including fresh weight, dry weight (Figure 5a,b), or dry matter percentages (Table 1). 

**Figure 4:**
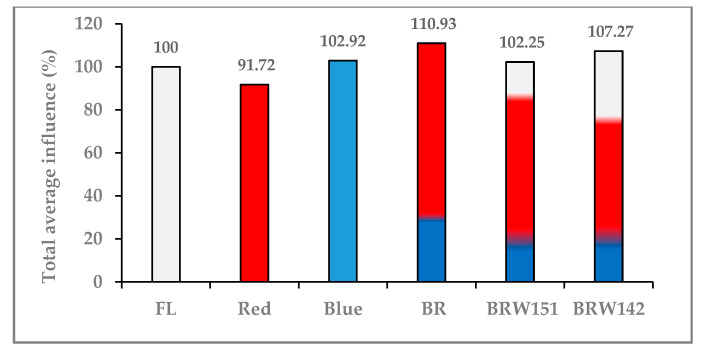
Total average of influence of different lighting treatments on the growth and morphological traits of *A. roxbughii* at the acclimatization stage. FL: fluorescent lamp; BR: blue–red; BRW151: blue: red: warm white LED ratio 1:5:1; BRW142: blue: red: warm white LED ratio 1:4:2.

As the above mentions, the obtained results indicated that the material biomass, including the stem cell anatomy of *A. roxburghii* showed broad variation responses under the different LED light treatments. The relative influence percentage of LED lights on each morphological and development indexes compared to FL were theoretically transformed from the average value when the value of each index of FL was assumed to be 100% (Supplement Data Table S2). Then, the total average of influence of each LED light on *A. roxburghii* plants was calculated from nine transformed data. Interestingly, we have found that the highest total average influence was in the BR treatment, while a slight inhibition was in the red treatment. All other light treatments showed negligible effects compared with the FL (Table 1; Figure 4).

**Figure 5:**
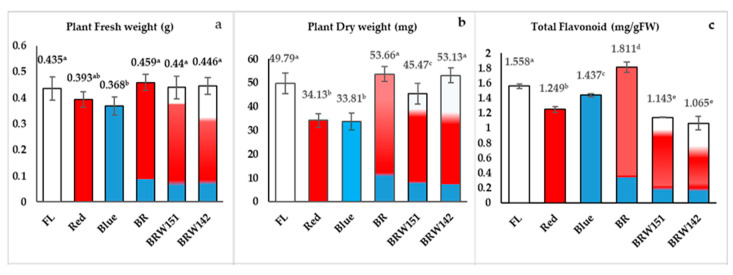
Effect of light conditions on *A. roxburghii.* Fresh weight (**a**); dry weight (**b**), and (**c**) total flavonoid accumulation. Error bars indicate standard deviations of three independent experiments. Different letters on bar graphs indicate the significant differences between treatment conditions by using Duncan’s multiple range test at *p* < 0.05.

For the plant fresh and dry weight, it was observed that BR, BRW151, and BRW142 were slightly enhanced but not significant to compare with the control (FL), while the blue light and red light caused a reduction in the fresh weight (Figure 5a). Similarly, BR and BR142 exerted slight enhancement of the plant dry weight, whereas significant dry weight reduction was found in red, blue, and BRW151 treatments as compared with the control (FL).

### 2.2. Effects of LED Lights on Total Flavonoids Accumulation

Our results showed that not only wavelength type, but the ratio also influenced flavonoid accumulation in *A. roxburghii*. The red wavelength caused a decrease in the total flavonoid contents by 1.25 mg/g, compared to the control (1.56 mg/g) (Figure 5c). Meanwhile, the blue wavelength was caused by a slightly negative influence on flavonoid biosynthesis in this plant. However, the combination of blue and red light in BR significantly provoked the percentage of total flavonoid in *A. roxburghii* plants, about 1.81 mg/g compared to 1.56, 1.25, and 1.44 mg/g in the FL, red, and blue LEDs, respectively (Figure 5c). On the other hand, the BRW142 induced the lowest total flavonoid contents (1.06 mg/g) compared to the control, followed by BRW151 (1.14 mg/g), red light (1.24 mg/g), and blue light (1.43 mg/g), while BR strikingly exerted the highest flavonoid contents by 1.81 mg/g. Overall, among all LEDs treatments, BR light has significantly enhanced the flavonoid contents than other light treatments.

### 2.3. Effects of LED Light on Expression Level of pal, chs, chi, and fls Encoding Genes

**Figure 6:**
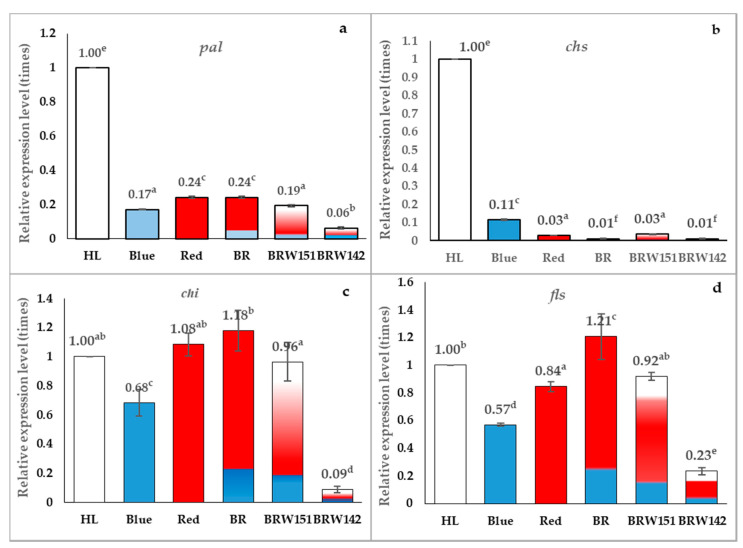
Effect of light conditions on the expression of several flavonoid biosynthesis-related genes: (**a**) *pal* (phenylalanine ammonia-lyase), (**b**) *chs* (chalcone synthase), (**c**) *chi* (chalcone isomerase), and (**d**) *fls* (flavonol synthase). Relative expression levels were determined by using the 2(-Delta Delta C(T)) method in fluorescent conditions as the control and *actin*2 as a housekeeping gene. Error bars indicate standard deviations of three independent experiments. Different letters on bar graphs indicate the significant differences between treatment conditions by using Duncan’s multiple range test at *p* < 0.05.

The expressional level of genes encoded for *pal*, *chs*, *chi*, and *fls* proteins were examined to further evaluate the effects of LED lights on the flavonoid accumulation in *A. roxburghii*. The obtained results in this study indicated that although the expression of *pal* and *chs* genes in all experimental conditions of LED lights were declined as compared to the control, the total flavonoid content in plants under BR LED was still higher than that in other lighting treatments (Figure 5c). The results showed that the transcription of *chs* of plants developed under all of the LED lights was extremely lower than that in the FL at the collected time (which is under the daylight period) (Figure 6b). 

This study showed that the expression of *chi* in *A. roxburghii* grown under BR treatment was about 1.18 times higher than the FL (Figure 6c). The results also indicated that the relative expressional level of *fls* in *A. roxburghii* grown under BR treatment was more strongly expressed than those under the FL (expression level was approximately 1.21 times higher) (Figure 6d). 

## 3. Discussion

Light is one of the most indispensable factors for plant growth and development, which is directly involved in the regulations of plant morphogenesis, metabolism, gene expression, photosynthesis, and other physiological responses. In the last decade, numerous studies have investigated the effects of light quality and light intensity on plant growth and functional chemical yields [40]. In this study, a high-value medicinal *A. roxburghii* plant was selected to investigate whether the different LED lights treatments and combined light conditions can accumulate or suppress on some main growth and morphological characteristics of *A. roxburghii* including plant height, stem diameter, number of leaves and roots, root length, leaf area, cell width, cell length, and dry matter. The fluorescent lamp (FL) was used as the control because FL often shows a wide frequency wavelength ranged from 405 to 579 nm and was reported to both promptly and greatly enhance the growth of many plant species [41]. Our LEDs and combined lights were accurately designed following conditions: mono-wavelength (1) red (630 nm); (2) blue (465 nm), and mixed-wavelength; (3) BR (20% B light and 80% R light); (4) BRW 151 (13.8% B light, 72.4% R light, and 13.8% white light); and (5) BRW142 (13.8% B light, 58.6% R light, and 27.6% W light). 

Plant species show different responses to light quality through the photoreceptors system. Generally, red and blue light exert the greatest effects on plant growth. For instance, the plant height of *Salvia miltiorrhiza* and *Tagetes erecta* was more stimulated under the blue light than red and fluorescent white light condition [42]; meanwhile, the red light was exerted a suppressive impact on leaf area, fresh weight, and plant height of cucumber seedlings [43]. Nevertheless, the leaf area of *Alternanthera brasilianna* was significantly enlarged [44] but induced shorter stems and roots of *Stevia rebaudiana* seedlings under the blue light treatment [45]. Contrarily, the red light was reported to significantly stimulate the height and weight of *Rehmannia glutinosa* and *Taraxacum officinale* [46,47]. The phenomenon of the different growth responses to red or blue light reflects the ability of plants to reciprocate changes in the light environment to complete their life cycle, and also due to the different action different actions of phytochrome and cryptochrome photoreceptors [4,47,48,49]. Moreover, cryptochromes and phototropin are either sensitive to blue or red wavelengths [50]. Most research showed that mono-wavelength might cause several drawbacks to a plant’s development. Therefore, in this study, in order to maintain the balance between regular developments and secondary compounds accumulation, the different wavelengths were combined.

In this present study, firstly, we have observed the differences in growth and morphological characteristics of *A. roxburghii* explants cultured in vitro among the different light and combined light conditions. Except for the increase in plant height, the mono-wavelength of blue and red light was significantly inhibited the growth biomass of *A. roxburghii* plant from comparing with the control (FL) (Figure 5a,b). Our result generally agreed with the recent report of Ye et al. [4] that the stem of *A. roxburghii* was shorter under the blue light treatment. The highest cell width was found under the blue treatment, while red light inhibited the cell width (Figure 2; Table 1). In contrast, red light had significant enhancement of the cell length, but the blue light was inhibited. Our results were consistent with some previous results revealed that red light (620–630nm) greatly affected plant elongation. Numerous plant species showed skotomorphology, when cultured under red light (630 nm), which could be seen with weak and elongated stem [51]. Not only affected plant stems but also the blue and red lights suppressed the development of *A. roxburghii* leaves. These results disclosed that the mono-wavelength (630 nm) of red light caused an extremely adverse influence on the stem’s morphology. With the combination of blue, red, and white light, the balance was recovered; abnormal morphologies were reduced. Interestingly, our findings found that the BR showed significant enhancement in both growth and biomass accumulation (Table 1; Figure 5a,b) 

Light conditions strongly affected plant metabolism, especially the accumulation of polyphenol compounds including flavonoids. The combination of blue and red light in BR significantly improved the percentage of total flavonoid in *A. roxburghii* plants, about 1.81 mg/g compared to 1.56, 1.24, and 1.43 mg/g fresh leaves in the FL, LED red, and LED blue, respectively. The reason might be the factors involved in the flavonoid biosynthesis pathway were regulated by both blue and red wavelengths. Liu et al., 2019, indicated that blue or red light had opposite effects on different flavonoid compounds in *Citrus sinensis* Osbeck. Meanwhile, the blue light promoted Diomestin 6-8-di-C-glucosie, Neoeriocitrin, Apigenin-6,8 di glucosise, Narirutin; red light enhanced the accumulation of Diosimin, Rhoifolin-4′-O-glusoside, Eriocitrin, Didymin, and Hesperidin [52]. On the other hand, the supplement of white diodes in the LED matrix caused the decline of total flavonoids in plants cultured under BRW151 and BRW142, compared to the control and BR lights (Figure 5c). Besides blue or red wavelengths, there was the presence of yellow and green wavelengths in these lamps, which were initially from white light emitting diodes in the matrix. Ye et al., 2017, showed that yellow light (generated with a light filter) led to a decrease in total phenolic compounds and flavone in *A. roxburghii* [4]. Likewise, Wang et al. (2018) also found that the lowest total flavonoid content was in the formula yellow light, and the total polyphenol content was the lowest in the green light treatment [31]. Consequently, the total biomass of plants slightly affected to compare to BR light (Figure 5a,b). These phenomena might be influenced by the effects of the white LED ratio in BRW142 lamps (approximate 28%) compared to BRW151 (about 14%). In addition, the reduction in red diodes in this lamp also might be the reason (about 58% red diodes) as compared to the BR lamp with up to 80% red diodes.

Phenylalanine ammonia-lyase (*pal*) encoded by the *pal* gene is the first enzyme in the phenylpropanoid pathway. It involves the synthesis of both polyphenols and lignin in plants. Numerous studies showed that the *pal* gene might be induced by light [53]. However, the transcription is also controlled by many transcription factors (TFs) involved in the lignin synthesis in plants, for instance: MYB TFs (AtMYB46, AtMYB 83, and MYP156) [4,54]. Previous works have also shown that there might be a regulatory mechanism existed in order to ensure the balance between lignin and flavonoid synthesis in plants. RNAi mutation of lignin synthesis in Arabidopsis resulted in not only reduced plant growth but also caused the improvement of flavonoid synthesis. This phenomenon could be reversed by decreasing the synthesis of flavonoids mediated via silencing chalcone synthase encoding gene (*chs*) [55]. Therefore, it can be seen that the expressional *pal* level may not significantly affect on the flavonoid accumulation, which might be controlled by other key enzymes at different branches in the pathway. The results obtained in this research showed that, although the expression of *pal* gene in all experiment LED lights were declined as compared to the control, the total flavonoid content in plants under BR LED was still higher than that in other lighting conditions (Figure 5c).

Besides *pal*, chalcone synthase (*chs*) is also involved in the polyphenol synthesis pathway, which synthesized chalcone naringenin backbone from a molecule of CoA-ester type of cinnamic acid (e.g., coumaric or ferulic acid) and three molecules of malonyl-CoA. Many previous studies have shown that *chs* gene can be induced by both biotic and abiotic stresses such as light/UV light or by circadian rhythms. Thain et al. [56] reported that the active rhyme of *chs* gene was different from other genes related to photosynthesis, especially genes coding for enzyme chlorophyll a/b-binding protein (CAB), which constituted the photosynthetic complex. *chs* expression occurred earlier than that of the *cab* gene, which transcriptional level usually was higher at the end of the dark cycle, just before the plant is exposed to light. Together with the previously published results, it has hypothesized that the expression of *chs* during the dark period might be a preparation for the polyphenol accumulation, which will be utilized by plants in the daylight [56]. In this study, we found that, at the transcription level, the *chs* gene expression in *A. roxburghii* grown under different LED conditions was lower than that under FL (Figure 5b). This might be influenced by the sample collected time (daytime), since the *chs* gene was only high expression at the end of the dark cycle.

The chalcone naringenin formed by the *chs* enzyme is an open-chain and needs to be closed by the chalcone isomerase (*chi*) enzyme. Therefore, the transcription, as well as the enzyme activity of *chi*, play an essential role in the synthesis of the basic aglycone of flavonoids after which this product will be used to synthesize different flavonoid branches by responsive genes evaluated the correlation between *chi* mRNA accumulation and anthocyanin concentration in *Ophiorrhiza japonica*. The authors reported that transcription of *chi* is one of the factors that directly affected the anthocyanin content in *Ophiorrhiza japonica*. This hypothesis is also supported by the previous study, which inactivated or increased the *chi* expressional level led to changes in anthocyanin and flavonoid contents in many plant species [57]. Besides, the *fls* gene also plays a vital role in flavonoid biosynthesis. *fls* encodes for flavonol synthase enzyme, which catalyzes the reaction transforming Dihydrokaempforol into Kaempferol (the major aglycon in *A. roxburghii* flavonoids) [4]. Figure 6c,d showed that among tested lights conditions, BR LED showed the highest expressional level in both *chi* and *fls* gene (respectively, 1.18 and 1.21 times compared with FL). This might be one of the main reasons for the increasing of flavonoid contents in *A. roxburghii* cultured under BR LED light in comparison with those under FL or other LED lights (Figure 5c).

Biological rhythms work based on the participation of many environmental induction factors, including light-sensing receptors. In particular, these receptors are affected by the lighting cycle as well as the characteristics of the lighting spectrum [58,59,60]. In this experiment, the different lighting conditions could influence the activity of those receptors and indirectly impact the activity of some genes that are controlled by circadian rhythms. However, further studies are required to reveal the correlation between light spectrum, light receptors, biological rhyme, and the flavonoid synthesis process in *A. roxburghii*. 

As the world attempts to exploit artificial lights to promote the secondary metabolites of vegetables and herbs, the big challenges remain regarding what the optimal time of the growth period of specific each plant under the optimal light intensity is and what wavelength light and how long irradiation of light should be completed [61]. Further research should be focused on the multiple adjustable combinations of LEDs in the different growth stages of the plant with varying light intensity, quality, and duration.

## 4. Materials and Methods

### 4.1. Plants Materials and Growth Conditions

Two-month-old *A. roxburghii* rooted in vitro plants (with 2 roots, 3 leaves, and about 3 cm in height) were used as the initial materials. Plants were cultivated in hydroponic pots (17 cm long, 12 cm wide, and 7 cm deep) supplemented with 1/10 strength macro and minor salt of Murashige and Skoog (1962) medium. The LED light source was placed at the top of the steel frame structure of the cultivation rack. The distance between the plant canopy and LED lights fluctuated from 40 to 60 cm. The silver shading materials were used to insulate in the different treatments. The relative humidity in the growth room (2m in length × 0.6 m in width) was 80%, and the temperature was in the range of 22–24 °C with 12 h of lighting. The plants were subjected to different LED light for 2 months under following conditions: mono-wavelength (1) red (630 nm), (2) blue (465 nm), and mixed-wavelength, (3) BR (20% B light and 80% R light), (4) BRW 151 (13.8% B light, 72.4% R light, and 13.8% W light), and (5) BRW142 (13.8% B light, 58.6% R light, and 27.6% W light). Fluorescent lamp was a multiple wavelength and was used as the control (Supplementary Figure S1). All the LED lights, mixed LED lights were ordered as accurate above conditions and designed by Dien Quang Lamp Join Stock Company, Ho Chi Minh city, Vietnam. The average of light intensity of the distance from the LEDs to the plant canopy was about 30 ± 2.13 µmol.m^−2^ s^−1^ and measured by the LI-COR LI-250A Light Meter (Li-Cor Biosciences, Lincoln, NE, USA). 

### 4.2. Morphological and Biomass Parameter Analyses

For measurements of the morphological characteristics, 30 random plants were selected from each treatment. Plant height, stem diameter (3rd node), leaf numbers, leaf area, root length, root numbers, fresh weight, and dry weight were measured. Briefly, the fresh weight was determined by using the electronic balance (Vibra, Japan) and dried to a constant weight under 70 °C before dry weight measurement. The stem diameter was recorded using the digital vernier caliper. The root length was measured from the top of the root tip to the main stem. Leaf area was measured by using image analysis method with ImageJ software [62]. The values shown in Table 1 are the mean of 3 replicates, with the 30 random samples for each treatment.

### 4.3. Stem Anatomical Characteristics

The second node of each plant was used to determine stem cell width and length. The 2nd node was excised from the stem and sectioned by using CS-05W Crimping cross-section monitor (Ushiyama Denki, LTD). Cross and longitude sections were directly observed under DN-200 Digital Microscope. Cells width and length were measured by the Scope Image 9.0 software (by Bioimager, Inc.). Values shown in Table 1 are the mean of 03 replicates; each replication contains the samples from 5 plants in each treatment with 5 sections/node/plant.

### 4.4. Determination of Total Flavonoids Contents

The total flavonoid content was determined by the colorimetric method of Marinova et al., 2005, and Sultana et al. [63,64]. The principle of the method is based on the coloration of flavonoid compounds with AlCl_3_ in an alkaline medium. One gram of fresh leaf sample was crushed homogeneously in liquid nitrogen, then extracted with absolute ethanol at the ratio of 1:3. The sample was further extracted on an ultrasonic machine for 30 min, then centrifuged to recover the ethanol solution. The extracted ethanol obtained from 3 repeats were combined and adjusted to equal volumes (10 mL) with ethanol. We shook well the mixture of 1 mL of the obtained extract, 4 mL of water and 0.3 mL of 5% NaNO_2_, left the mixture at room temperature for 5 min, then added 0.3 mL of 10% AlCl_3_. After 6 min, we added 2 mL of 1 M NaOH solution, mixed well, and adjusted the volume with water to 10 mL. The absorbance of the mixtures was recorded at 510 nm by UV/VIS Spectrophotometer Camspec M108. The total flavonoid contents were calculated based on the quercetin calibration curve, performed under the same conditions. The results were expressed in mg/g of fresh leaf [65].

### 4.5. Expression Analysis of Several Flavonoid Biosynthesis-Related Genes

Three samples of each light condition were used for total RNA extraction. The third leaves of 2-month-old plants were collected at the same time, after 6 h exposed to the light regime in a daily 12 h light cycle. Total RNA was isolated from *A. roxburghii* leaves by using TRizol^TM^ Reagent (Invitrogen^TM^) following manual instruction and then transcribed to cDNAs using RevertAid First Strand cDNA Synthesis Kit (ThermoFisher Scientific). The expressional levels of flavonoid biosynthesis-related genes (*pal*, *chs*, *chi*, and *fls*) together with *actin*2 as housekeeping gene was determined by real-time PCR system (Rotor-Gene Q, QUIAGEN). PCR was conducted by using SYBR Green (SsoAvanced Universal SYBR Green Supermix, BioRad) by following manufacturing instruction. PCR thermal program was performed as following: pre-denature 95 °C/3 min; 40 cycles of denature 95 °C/10 s; annealing 57 °C/20 s; extension 72 °C/20 s. DNA melting curve was determined by ramp heating from 65 °C to 95 °C and holding within 5 s at 95 °C for the analysis. The different relative level of gene expression between lighting conditions was determined based on the 2(-Delta Delta C(T)) method [66]. The experiment is considered to be different when processing ANOVA statistics of 03 replications with *p*-value < 0.05. Specific primers for analysis were described by Yang et al. [67]; Zhang et al. [68], and Zhang et al. [69,70]. The specific gene primers involved in flavonoids biosynthesis-related genes expression are presented in Supplementary Table S1.

### 4.6. Statistical Analysis

StartGraphic XV (Statpoint Technologies, Inc., Warrenton, VA, USA) was used for all statistical analyses. Data were analyzed using one-way ANOVA analysis. Differences between the means of each treatment were detected by using Duncan’s multiple range test with *p* < 0.05.

## 5. Conclusions

In summary, light source influenced not only plant growth and morphogenesis but also metabolites accumulation. This paper showed that *A. roxburghii* required both blue and red wavelengths for normal development. Plants grew under BR light (1 blue diodes with 4 red diodes) obtained the highest stem diameter, leaf area, and biomass. Especially, BR light could promote the accumulation of flavonoid properly due to enhancing expression of several critical genes involved in the flavonoid biosynthesis pathway. This paper provides an effective method that uses LED light to improve the development and flavonoid accumulation in *A. roxburghii*.

## Figures and Tables

**Table 1:** Influence of different lighting conditions on morphology and flavonoid contents of *A. roxbughii* at the acclimatization stage.

Light Conditions	FL	Red	Blue	BR	BRW151	BRW142
Average of plant height (cm) *	6.58 ^a^ ± 0.38	7.36 ^c^ ± 0.92	7.23 ^c^ ± 0.5	7.18 ^bc^ ± 0.27	6.65 ^a^ ± 0.42	6.99 ^abc^ ± 0.78
Average of stem diameter (mm) *	16.7 ^cd^ ± 2.57	11.4 ^a^ ± 1.39	15.6 ^c^ ± 2.77	17.6 ^d^ ± 1.99	16.9 ^bc^ ± 2.02	16.6 ^cd^ ± 1.8
Number of leaves per plant *	5.13 ^a^ ± 0.35	5.06 ^a^ ± 0.59	5.53 ^bc^ ± 0.51	5.87 ^c^ ± 0.63	5.33 ^ab^ ± 0.48	5.8 ^c^ ± 0.56
Cell width of 2nd node sections (µm) **	28.58 ^c^ ± 3.2	25.37 ^a^ ± 4.4	36.87 ^b^ ± 3.1	30.31 ^d^± 3.7	33.11 ^e^ ± 3.6	27.64 ^bc^ ± 3.8
Cell length of 2nd node sections (µm) **	35.94 ^bc^ ±5.6	44.33 ^e^ ± 5.5	31.23 ^a^ ± 3.9	44.2 ^e^ ± 7.4	31.98 ^ab^ ± 5.1	37.82 ^cd^ ± 8.5
Leaf area (cm^2^) *	3.36 ^c^ ± 0.34	1.99 ^a^ ± 0.32	3.02 ^bc^ ± 0.43	4.67 ^d^ ± 0.89	3.61 ^c^ ± 0.36	3.37 ^c^ ± 0.7
Number of roots per plant *	1.73 ^a^ ± 0.45	2.00 ^ab^ ±1.04	2.06 ^bc^ ± 0.25	1.8 ^ab^ ± 0.41	1.93 ^ab^ ± 0.25	2.26 ^cd^ ± 0.45
Average of roots length (cm) *	2.94 ^bc^ ±0.43	2.47 ^a^ ± 0.54	3.24 ^c^ ± 0.47	2.8 ^ab^ ± 0.45	2.94 ^bc^ ± 0.49	3.23 ^c^ ± 0.34
Dry matter percentages (%) *	11.45	8.68	9.18	11.69	10.33	11.91

FL: fluorescent lamp; BR: blue–red; BRW151: blue: red: warm white LED ratio 1:5:1; BRW142: blue: red: warm white LED ratio 1:4:2. Different letters (^a,b,c,d,e^) within a row express significant differences between treatment conditions by using Duncan’s multiple range test at *p* < 0.05; * values indicate mean ± SE of 03 replications experiment, each experiment including 30 two-month-old *A. roxburghii* explants; ** values indicate mean ± SE of 03 replications experiment, each replication contains samples from 5 plants each treatment with 5 sections/node/plant.

## Supplementary Materials

The following are available online at https://www.mdpi.com/2223-7747/9/10/1344/s1, Table S1: Primers information used for flavonoids biosynthesis-related genes expression analysis. Table S2: Total average of influence (%) of different lighting treatments on the growth and morphological traits of A. roxbughii. Figure S1: Characteristics of the respective LED irradiance spectra in the different treatments.
