# Supplementary material for: LED Lights Promote Growth and Flavonoid Accumulation of Anoectochilus roxburghii and Are Linked to the Enhanced Expression of Several Related Genes
Source: Plants (Basel). 2020 Oct 12;9(10):1344. doi: 10.3390/plants9101344 (PMC7599663; doi:10.3390/plants9101344)
Supplement: Supplementary file 1 [file plants-09-01344-s001.pdf]

Table 1S. Primers information used for flavonoids biosynthesis-related genes expression analysis

Gene name	Sequence (5′ → 3′)	Size (bp)
pal	F: CGAGGAACAAGGCCATCCAT	105
	R: GCGAACATGAGCTTCCCGAT	
chs	F: AGGCGTTCACTCCCTTTAGGC	135
	R: ACGTGCCTGGAAGTGGTTAG	
chi	F: AAGGTGACGTTCCTCGTTCC	~220
	R: CTGCGGTGAATGGGTGTAGA	
fls	F: GGCGGAGAAGGAGAAGTACG	~190
	R: GGCGGCCAGACATTATGGAA	
EF1	F: GATGACCGGGAGCATCAATGAC	296
	R: CTGAGCGTGAACGTGGTATCAC	

Table 2S. Total average of influence (%) of different lighting treatments on the growth and morphological traits of *A. roxbughii*

**Light conditions**	**FL**	**Red**	**Blue**	**BR**	**BRW151**	**BRW142**
Average of plant height	100	111.85	109.88	109.12	101.06	106.23
Average of stem diameter	100	68.26	93.41	105.39	101.20	99.40
Number of leaves per plant	100	98.64	107.80	114.42	103.90	113.06
Cell width of 2nd node sections	100	88.77	129.01	106.05	115.85	96.71
Cell length of 2nd node sections	100	123.34	86.89	122.98	88.98	105.23
Leaf area	100	59.23	89.88	138.99	107.44	100.30
Number of roots per plant	100	115.61	119.08	104.05	111.56	130.64
Average of roots length	100	84.01	110.20	95.24	100.00	109.86
Dry matter percentages	100	75.81	80.17	102.10	90.22	104.02
**Total average of influence**	**100**	91.72	102.92	110.93	102.25	107.27

Note: Relative influence percentage = (100 x Average value under LED light)/ Average value under FL light.

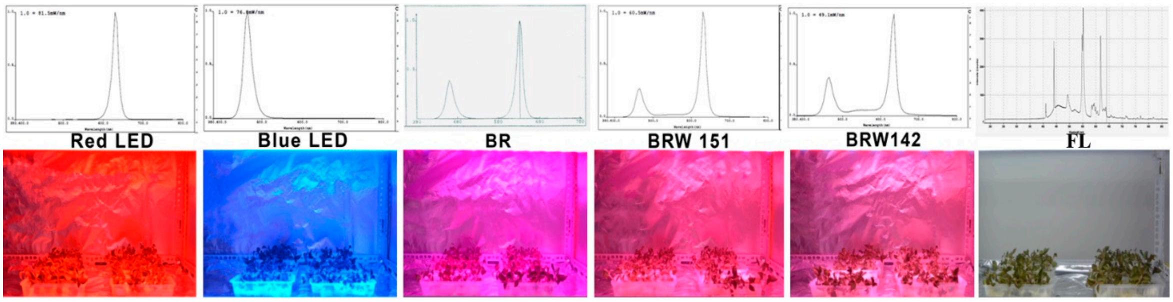

Figure 1S. Characteristics of the respective LED irradiance spectra in the different treatments
